# Editorial: Endocrine and metabolic consequences of childhood obesity

**DOI:** 10.3389/fendo.2022.1000597

**Published:** 2022-08-25

**Authors:** Dénes Molnár, Artur Mazur, Aneta Monika Gawlik, Grzegorz Telega, Elpis Vlachopapadopoulou, Malgorzata Wojcik

**Affiliations:** ^1^ Department of Pediatrics, Medical School, University of Pécs, Pécs, Hungary; ^2^ National Laboratory for Human Reproduction, Szentágothai Research Centre, University of Pécs, Pécs, Hungary; ^3^ University of Rzeszov, Rzeszov, Poland; ^4^ Department of Pediatrics and Pediatric Endocrinology, Faculty of Medical Sciences, Medical University of Silesia, Katowice, Poland; ^5^ Medical College of Wisconsin, Milwaukee, WI, United States; ^6^ Panagiotis & Aglaia Kyriakou Children’s Hospital, Athens, Greece; ^7^ Department of Pediatric and Adolescent Endocrinology, Chair of Pediatrics, Pediatric Institute, Jagellonian University Medical College, Krakow, Poland

**Keywords:** obesity, children, childhood obesity, metabolism, endocrinology, insulin resistance, type 2 diabetes

During a very short period of time (approximately four decades), obesity has become a global epidemic and an urgent health and economical burden due to its impact on public health and on the whole society. Obesity is a complex multifactorial disease defined by excessive adiposity and is linked to an increased risk for many noncommunicable diseases (NCDs). Overweight and obesity affect almost 60% of adults and nearly one in three children (29% of boys and 27% of girls) in the European Region according to the latest report of the World Health Organization (1). Childhood obesity is associated with early metabolic sequelae and also linked to increased risk of persistent obesity in adulthood and long-term complications. Obesity increases the risk of the development of the metabolic syndrome, cardiovascular disease, childhood-onset type 2 diabetes mellitus and its associated retinal and renal complications, non-alcoholic fatty liver disease, obstructive sleep apnea, premature menarche, polycystic ovary syndrome, infertility, asthma, orthopedic complications, psychiatric disease, and increased rates of malignancies. Obviously, the present Research Topic could not address all the above listed consequences but rather focused on selected endocrine and metabolic aspects. In this timely Frontiers Research Topic, researchers practicing world wide and having different disciplines contributed reviews (two narrative and one systematic), and novel data on the challenges obesity presents in attempts to stimulate debate on ways forward.

One of the main threads of the papers published in this Research Topic is insulin reistance and metabolic syndrome. Some papers searched probable markers or predictors of insulin resistance. Anthropemtric measures and indexes are cheap, and widely used as screening tools for overweight/obesity. There are further ambitions to use these anthropometric measures and indexes to predict the presence or the future development of metabolic and hormonal complications linked to obesity. DeLacy and Josefson in their mini-review investigated the power of different anthropometric measures and indexes in the prediction of the development of future insulin resistance. The conclusion, as it was expected, is that there is no adequate evidence that the different anthropometric measurements and indexes have sufficient strength to predict the development of future insulin resistance.

Wang et al. investigated the role of tri-ponderal index (TMI) [weight (kg)/ height^3^ (m^3^)] in the screening of metabolic syndrome. This study included large number of children (57,20 Chinese and 10,441 American), proposed reference values for TMI applying sophisticated statistical analyses. The authors suggested that the TMI could be an accurate and convenient, population-based screening tool for metabolic syndrome and metabolic risk factors in children and adolescents. However, we are not convinced that TMI is superior to BMI or other anthropometric indexes.

Iwani et al. investigated triglyceride/HDL-cholesterol ratio (TG:HDL-C) as a posible marker of insulin resistance in 524 children aged 10-16 years. Since fasting blood glucose level is not a reliablile marker of impared glucose homeostasis in children they recommend the replacement of fasting blood glucose by TG:HDL-C ratio as a better surrogate marker of insulin resistance in children. This is an interesting hypothesis that need further confirmation.

Hyperuricemia is strongly related to obesity and may be linked to insulin resistance, type 2 diabetes and increased cardiovascular risk. Niu et al. investigated the associations between serum uric acid, insulin resistance (defined by homeostasis model assessment-insulin resistance, HOMA-IR) and BMI in 369 Chinese children aged 4-17 years with obesity in a retrospective, cross-sectional design. Due to the design causal relationship between hyperuricemia and insulin resistance could not be established, however, the results suggest that serum uric acid level may have a mediator role in the development of obesity-induced insulin resistance in children and adolescents.

Non-alcoholic fatty liver diesease (NAFLD) is prevalent among children with obesity. The gold standard in diagnosing NAFLD and liver fibrosis is liver biopsy, which is an invasive technique. Several studies have analyzed non-ivasive markers (ultrasound elastography, liver transaminase levels, TG:HDL-C ratio, HOMA-IR, etc.) of liver steatosis and fibrosis. Furthner et al. investigated the possible role of the single point insulin sensitivity estimator (SPISE) as a marker of NAFLD in children with obesity. They used sophisticated, modern methods (magnetic resonace imaging, hyperinsulinemic clamp test) and concluded that SPISE can be a useful surrogate marker of hepatic insulin resitance and NAFLD in obese children.

It is well-known that secondary alterations in steroid metabolism are present in children with obesity. In the paper by Suminska et al. the relationship between obesity, insulin resistance and steroid metabolism was resurrected. The results are not powerful due to the relatively low sample size and the fact that enzyme activities were estimated by the ratios of different metabolites in the collected urine samples. Although, the paper directs our attention towards steroid metabolism in childhood obesity, especially in girls.

Asprosin is a recently identified glucogenic adipokine stimulating hepatic glucose release. Its physiologic role has not yet fully clarified. Asprosin serum levels increase in fasting conditions and decrease after refeeding. Corica et al. reported a paradoxical increase of asprosin in a subroup of children with obesity. The clinical significance of this altered asprosin response to oral glucose load needs further clarification.

There is evidence that metabolic syndrome and cardiometabolic abnormalities, are already present in children and adolescents with obesity. It is especially alarming that the number of abnormal cardiometabolic parameters rose markedly over the study period (2008-2017) in a large cohort of Chinese children and adolescents from the study of Wang et al

Another thread in this Research Topic deals with different aspects of circulating leptin levels (three original research papers). Brandt et al. studied the circulating leptin levels in children with obesity and fatty liver disease. Leptin levels are generally higher in children with obesity as compared with normal weight counterparts. Brandt et al. reported an intriguing observation: children with sonographic steatosis hepatis had significantly lower z-scores of circulating leptin levels compared to children with normal liver ultrasonography. Whether children with NAFLD and low leptin levels (partial leptin deficiency) should be considered as a special subgroup needs to be further explored. Moreover, a crucial question is whether these children could benefit from leptin treatment. An interesting study was published by Adamczewska et al. who investigated the relationship between leptin and TSH levels in obese short children. It is an important finding highlighting that children with idiopathic short stature and obesity may deserve special attention, since in this subgroup leptin does not increase TSH secretion.

Previous studies have revealed that serum 25(OH)D concentrations show a strong inverse correlation with fat mass and serum leptin levels, however, causal relationship has not been confirmed. Nevertheless, the risk of vitamin D insufficiency is 3 times higher in obese children compared to normal weight counterparts. The results of Khwanchuea and Punsawad are more or less in line with previous findings. Due to the cross-sectional design the causal link between body fat, leptin and vitamin D levels could not be established.

There are two papers in the Research Topic investigating the modulating effects of pubertal development and the effect of prepubertal BMI on early onset of puberty Hypoadiponectinemia is well-known phenomenon in obesity, however, the genetic, environmental and lifestyle factors and their crosstalk influencing adiponectin levels are not fully understood. The study of Wu et al. clearly demonstrated that puberty modulates the asociations between adiponectin, and genetic variants, lifestyle factors and gene-by-lifestyle interactions. The study included a large sample size and the results and the hypothesis of the authors are exemplarily demonstrated.

A continuous trend toward earlier onset of puberty has been observed around the world. During the same period, the prevalence of obesity has increased worldwide. The question whether prepubertal obesity (BMI) influences the onset of puberty was investigated by Fang et al. in an excellent study.

Children suffering from type 1 diabetes (T1D) usually were considered as lean, however, recent studies have shown that the prevalence of overweight/obesity is increasing in individuals with T1D. The overlap of obesity and T1D may lead to “double diabetes”, may hinder effective therapy and result in significant health consequences. The exciting and challenging topic of “double diabetes” was reviewed by Ciezki et al. in a really comprehensive way.

An excellent systematic review by Barros et al. invesigated the effects of overweight/obesity on motor performance in children. The review included 33 rigorously selected studies. The results confirmed and stregthened previous findings that obesity is associated with not only low motor performance but it also linked to increased physical health risk.

As guest editors of this Research Topic, we were rewarded by high quality contributions embracing many aspects of endocrine and metabolic consequences of childhood obesity. We do hope that the published papers will be useful reading for people working in basic and clinical sciences and also for those interested in public health. The aforementioned articles may help in unraveling new biomarkers and in unfolding crucial links between obesity and its consequences. Furthermore, they may stimulate and fertilize future research in this exciting scientific field.

## Author contributions

DM drafted this editorial. AM, AG, GT, EV and MW revised and approved the final submitted version.

## Conflict of interest

The authors declare that the research was conducted in the absence of any commercial or financial relationships that could be construed as a potential conflict of interest.

## Publisher’s note

All claims expressed in this article are solely those of the authors and do not necessarily represent those of their affiliated organizations, or those of the publisher, the editors and the reviewers. Any product that may be evaluated in this article, or claim that may be made by its manufacturer, is not guaranteed or endorsed by the publisher.
